# Highly efficient TiO_2_-functionalized nylon-6 nanofibrous membranes for rapid adsorptive removal of atrazine from water

**DOI:** 10.1039/d6ra02398c

**Published:** 2026-05-21

**Authors:** Saira Sidhu, Syeda Sara Hassan, Muhammad Rizwan, Zeeshan Khatri, Safina Kamboh, Akbar Ali, Khalid Hussain Thebo, Ahmed Nadeem

**Affiliations:** a U.S. – Pakistan Centre for Advanced Studies in Water, Mehran University of Engineering & Technology Jamshoro Pakistan sshassan.uspcasw@faculty.muet.edu.pk; b Department of Textile Engineering, Mehran University of Engineering & Technology Jamshoro Pakistan; c MIIT Key Laboratory of Critical Materials Technology for New Energy Conversion and Storage, State Key Laboratory of Urban Water Resource and Environment, School of Chemistry and Chemical Engineering, Harbin Institute of Technology Harbin 150001 PR China; d Department of Chemistry, Mirpur University of Science & Technology (MUST) Mirpur A&J Kashmir Pakistan khalidthebo@yahoo.com; e Department of Pharmacology and Toxicology, College of Pharmacy, King Saud University Riyadh 11451 Saudi Arabia

## Abstract

Increasing concentrations of the atrazine pesticide in water pose a significant risk to human health and aquatic life. In this study, TiO_2_-functionalized electrospun nylon-6 nanofibrous membranes are developed for the adsorptive removal of atrazine under batch conditions. The as-synthesized nanofibrous composite membrane was characterized using a scanning electron microscope (SEM), X-ray diffraction (XRD), Brunauer–Emmett–Teller (BET), Fourier-transform infrared spectroscopy (FTIR), *etc.* The SEM studies revealed the average fiber diameter to be in the range of 110–130 nm. A BET analysis of the surface area of nylon-6/TiO_2_ (24.5 m^2^ g^−1^) was obtained with surface area and porosity. The XRD pattern confirms the crystallinity of materials and membranes. Further, the incorporation of TiO_2_ nanoparticles (NPs) also enhanced the tensile strength (1.2 MPa) of the composite membrane and its adsorption capacity. Then, liquid chromatography-tandem mass spectrometry (LC-MS/MS) was used to evaluate the removal efficiency of atrazine. Furthermore, the key adsorption parameters, such as pH, initial concentration of atrazine, contact time, nanofiber dosage, *etc.*, were also optimized. The as-prepared nylon-6/TiO_2_ nanofibrous membrane exhibited a good adsorption removal efficiency of 67.12 mg g^−1^ (as determined by the Langmuir isotherm model). This study suggests monolayer chemisorption behavior. The adsorption kinetics followed a pseudo-second-order (PSO) model, with equilibrium reached within approximately 30 minutes of contact time. In addition, the removal efficiency of the nylon-6/TiO_2_ composite membrane was also compared with both the pristine TiO_2_ and nylon membranes, and it showed significantly higher efficiency. We believe such a cost-effective, and energy-efficient nanofibrous membrane system can be an alternative solution for rapid wastewater treatment applications.

## Abstract 2

Herein, TiO_2_-functionalized nylon-6 nanofibrous membranes were fabricated and demonstrated excellent atrazine adsorption capacity up to 67.12 mg g^−1^.

## Graphical abstract

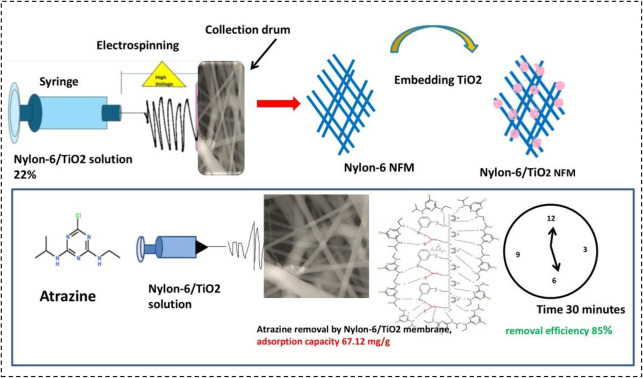

## 1. Introduction

Rapid urbanization and industrial revolutions have generated numerous environmental challenges. Among them, contamination of drinking water is one of the most pressing issues in the world. A wide range of industrial pollutants, including organic dyes, pharmaceutical residues, pesticides, and other micro-contaminations, are entering into water bodies and affecting the properties of water such as color, taste, and odor.^1,2^ In agricultural economies like Pakistan, the frequent use of organochlorine pesticides and herbicides has triggered serious ecological and public health consequences.^3^ Each year, more than 500 000 people suffer from pesticide poisoning in Pakistan, and the nation lacks monitoring data on pesticide usage, contamination levels, and its exposure. It is estimated that only 0.1% of pesticides serve their proposed purpose of pest control, while the remaining 99.9% contribute to environmental pollution.^4,5^ Such contaminants are difficult to remove from agricultural runoff and wastewater bodies due to their persistent nature.

Globally, the World Health Organization (WHO) reports nearly one million cases of acute pesticide poisoning annually, with mortality rates ranging from 0.5% to 2%.^6^ Pesticides applied to crops often persist in the soil and enter water bodies through irrigation runoff and rainfall, contaminating freshwater resources.^7^ With over 500 registered pesticide compounds and nearly 54 000 formulations, these substances are widely dispersed and chemically stable, often resisting biological and natural degradation mechanisms. The overuse and mismanagement of pesticides not only lead to bioaccumulation in food chains but also pose long-term health risks, including endocrine disruption, infertility, respiratory illnesses, and increased cancer risk.^8^ Approximately 2.5 million tons of pesticides are applied globally each year, and the trend is steadily increasing.^3^ Additionally, these chemicals are increasingly used in consumer products such as paints, plastics, and food packaging to inhibit the growth of pests, bacteria, fungi, and algae, further expanding their environmental footprint.^9^ In Pakistan, the absence of a centralized and updated pesticide usage database severely limits monitoring and enforcement efforts.^10^ Chronic exposure to low levels of toxic pesticides has been linked to serious health risks, including carcinogenesis, neurological disorders, and reproductive issues. Among the most persistent are *s*-triazine herbicides, such as atrazine, simazine, cyanazine, and propazine. Atrazine, widely used for controlling weeds in crops like wheat and sugarcane, is known for its environmental persistence, with degradation times ranging from 10 to 105 days in water and up to 385 days in soil.^11^ It is particularly harmful to aquatic life and has been linked to hormonal disruptions in humans.^12^ In response to the growing threat of water contamination, various non-conventional treatment technologies have been developed, including advanced oxidation processes (AOPs), reverse osmosis (RO), nanofiltration (NF), membrane bioreactors, adsorption using novel materials, and photocatalysis.^13–15^ While these methods show promise in degrading or removing complex organic pollutants, they are not without limitations. AOPs, for instance, require high energy input and precise operational conditions, and often generate secondary pollutants or toxic by-products. Reverse osmosis and nanofiltration systems, though effective, are costly to install and maintain, prone to membrane fouling, and generate brine waste that poses its own environmental disposal challenges.^16^ Photocatalysis and membrane bioreactors require expensive catalysts or membranes and are often less effective for compounds with low reactivity or high stability.^17^ Adsorption technologies, while simple and economical, are limited by low regeneration efficiency and adsorbent saturation.^18^ Moreover, these techniques may not fully degrade contaminants but only transfer them from one phase to another, *e.g.*, from water to solid adsorbents, creating further disposal issues. Many of these technologies also lack scalability and require technical expertise, making them less feasible for widespread use in rural or low-resource settings. To overcome these challenges, nanomembranes filtration has emerged as a cutting-edge of water treatment technique offering several advantages over traditional methods.^19^ Nanomembranes are ultrathin filtration membranes with nanoscale pores and functionalized surfaces that allow for high selectivity, low energy consumption, and efficient removal of micropollutants, including pesticides, heavy metals, pharmaceutical residues, and pathogens.^20^ Their customizable surface properties help reduce membrane fouling and enhance water flux, improving the operational lifespan of the system. Additionally, nanomembranes can operate under lower pressure conditions compared to conventional membranes, thus lowering energy demands. Their compact size and integration potential make them suitable for portable and decentralized water treatment systems, which is particularly beneficial in resource-constrained or remote areas. Furthermore, recent advances in nanocomposite materials such as graphene oxide,^21^ titanium dioxide,^22^ and carbon nanotubes^23^ have enhanced the mechanical strength, antimicrobial properties, and contaminant rejection performance of nanomembranes, paving the way for sustainable and scalable solutions. Despite some ongoing challenges related to cost and material stability, nanomembrane technology represents a promising frontier for tackling persistent organic pollutants like *s*-triazine herbicides in contaminated water systems.^24^

Herein, nylon-6/TiO_2_ nanofibrous membranes are fabricated through electrospinning method and used for the efficient removal of atrazine pesticide under batch conditions. The removal percentage of atrazine was quantitative measured by LC-MS/MS technique. Furthermore, several parameters such as key adsorption parameters, such as pH, initial concentration of atrazine, contact time, nanofiber dosage, *etc.*, were also optimized. The as-prepared nylon-6/TiO_2_ nanofibrous membrane exhibited good adsorption removal efficiency of 67.12 mg g^−1^ (as determined by the Langmuir isotherm model). This study suggests monolayer chemisorption behavior. The adsorption kinetics followed a PSO model, with equilibrium reached within approximately 30 minutes of contact time.

## 2. Experimental work

### 2.1. Chemicals and materials

High-purity chemicals were purchased from Sigma-Aldrich and Merck and were used without further purification for the preparation of nanofibrous composite membranes. The atrazine standard (99.7%), nylon-6 (CO–NH) polymers, nitric acid (65%), methanol (≥99.8%), titanium tetraisopropoxide (97%), *meta*-cresol, dimethyl formamide (99%), and formic acid (≥96%) were purchased and used without further purification.

### 2.2. Synthesis of TiO_2_ NPs by the sol–gel method

**Scheme 1:**
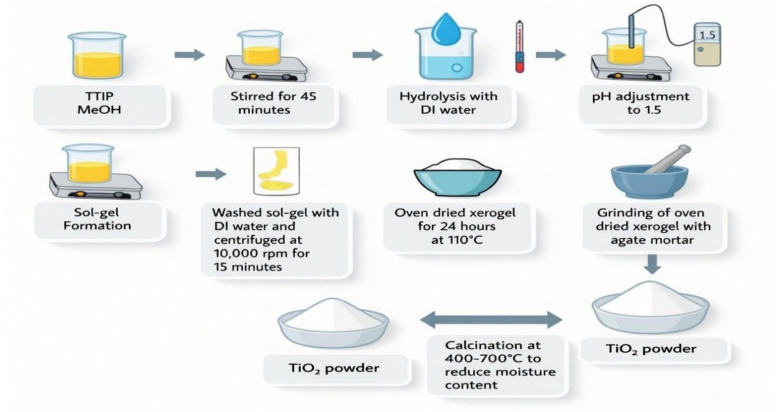
Schematic representation of the synthesis of TiO_2_ NPs using the sol–gel method.

First, a solution of 97% titanium tetraisoperoxide (TTIP) and methanol (≥99.8%) was prepared with a molar ratio of 1 : 15 (TTIP : MeOH) (Scheme 1). Then the solution was stirred for 45 min using a magnetic bar. Further, hydrolysis of TiO_2_ was carried out by using deionized (DI) water with a molar ratio of 1 : 4 (TTIP : DI water). The pH of the mixture was adjusted by adding nitric acid (65%) up to 1.5. Sol–gel formation was observed as a white colloid of TiO_2_ sol–gel after 24 h with continuous stirring. After that, the sol–gel mixture of TiO_2_ was washed with DI water and centrifuged at 10 000 rpm for 15 min. A white jelly-like gel was formed and dried in the oven for 24 h at 110 °C to remove moisture from the material. Then dried xerogel was ground using an agate mortar for 1 h. Finally, the dry TiO_2_ NPs were calcined at 400, 500, 600, and 700 °C for 4 h and labeled TiO_2_-400, TiO_2_-500, TiO_2_-600, and TiO_2_-700, respectively.

### 2.3. Fabrication of nylon-6/TiO_2_ nanofibrous membranes

**Fig. 1:**
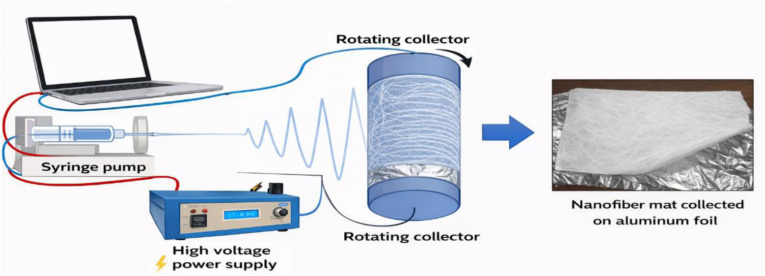
Experimental electrospinning setup for the fabrication of nylon-6/TiO_2_ nanofibrous membranes.

Nylon-6/TiO_2_ nanofibrous membranes were synthesized according to the as-prepared setup (Fig. 1). Nylon-6 polymer solutions were prepared by dissolving different concentrations (15, 20, and 25 wt%) of nylon polymer in formic acid. The mixture was stirred at 40 rpm using a magnetic stirrer for 2 h to ensure complete homogenization of solutions. As for the fabrication of composite membranes, first, a nylon-6 solution (22%, v/v) was prepared using a binary solvent system of formic acid and *meta*-cresol in an 80 : 20 (v/v) ratio. Then, both nylon-6 and nylon-6/TiO_2_ solutions were stirred for a further 12 h at room temperature before electrospinning to confirm complete uniform dispersion of NPs. Then, the nanofibrous membranes were prepared using a Nanocare Electrospinning System. The nylon-6/TiO_2_ precursor was loaded into a 20 mL syringe fitted with a stainless-steel needle. The syringe was mounted onto a precision pump, and the positive lead of the high-voltage power supply was connected to the needle tip. The grounding terminal was attached to the collector (receiver) to establish the electric field. The fabrication of both neat and composite nanofibrous was carried out under the following optimized parameters, such as 16 kV voltage, 16 cm distance between needle and collector, 0.100 mL min^−1^ flow rate, and drum rotation speed of 2.25 m min^−1^. The fabrication process was conducted under controlled conditions by varying one parameter at a time while maintaining the other constant.

### 2.4. Characterization techniques

The morphological and structural properties of the as-synthesized NPs and nanofibrous membranes were characterized using several techniques. UV-visible spectrophotometer (PerkinElmer UV/vis Lambda 365) confirmed the preparation of NPs by identifying characteristic absorption peaks at specific wavelengths. SEM (TESCAN, USA) was used to examine the surface morphology, size and shape of the membrane. Fourier transform infrared spectroscopy (FTIR, PerkinElmer Spectrum Two, USA) confirmed the functional groups, and zeta potential analysis (Zeta Sizer Nano-ZS, model no. MISS, Japan) measured the surface charge of the material. A XRD (Rotaflex RTP 1300 with Cu Kα radiation, *λ* = 1.5402 Å) was used to study the lattice structure and confirm the incorporation of TiO_2_ NPs into the nanofibers. Finally, elemental composition of material and membranes were studied with help of Energy Dispersive X-ray Spectroscopy (EDS) technique.

Then, LC-MS/MS was used for quantitative analysis of atrazine using positive electrospray ionization mode (ES^+^). LC-MS/MS (Waters ACQUITY UPLC system) coupled with a triple quadrupole MS/MS detector Xevo TQ-S (MASS HUNTER) was used for analysis of atrazine. The data acquisition and processing were collected with MassLynx software and chromatographic separation was achieved using ACQUITY UPLC BEHC C18 Column (1.7 µm, 2.1 × 100 mm). The data was obtained by using following LC conditions. Mobile phase A: 98 : 2 (v/v) H_2_O : MeOH + 0.1% formic acid (HCOOH) and mobile phase B : MeOH + 0.1% formic acid (with 10 mM ammonium formate in the aqueous component, as per method optimization). The flow rate was 0.5 mL min^−1^, the injection volume was 100 µL (full loop), and the column temperature was maintained at 40 °C. The mobile phase gradients program of the binary pump was 90% A and 10% B at the beginning for 0.25 min, followed by 90% B for 5 min and then 2% A and 98% B for 7.75 min. The mass spectrometer was operated in ESI^+^ mode; the interface current voltage was 0.6 kV, the heat block temperature was 150 °C, and the desolvation line temperature was 250 °C, with nebulizer nitrogen gas (150 L h^−1^) and drying gas of 150 L h^−1^.

### 2.5. Optimized parameters of kinetic studies

Different experiments were performed to study the adsorption and removal efficiencies and mechanism of atrazine pesticide by optimizing various parameters, including the effect of pH, varying concentrations, *etc.* First, the experiments were conducted at different concentrations of atrazine (from 100 to 1000 ppb), adsorbent dosage (2–10 mg), and contact times (30–150 min) at room temperature and neutral pH, with the solution stirred at 120 rpm to facilitate atrazine adsorption. A 50 mL atrazine solution was prepared at the desired pH and concentration, after which the adsorbent was added, and the mixture was shaken on an orbital shaker. Samples were collected and analyzed using a UV-vis spectrophotometer (PerkinElmer Lambda) with a deuterium–tungsten lamp (200–800 nm). The removal rate of the membrane was calculated using eqn (1)–(3): adsorption capacity at time *t* (*Q*_*t*_), at equilibrium (*Q*_e_), and removal efficiency (*R*).

1 *Q*_*t*_ = (*C*_0_ − *C*_*t*_)*V*/*M*

2 *Q*_e_ = (*C*_0_ − *C*_e_)*V*/*M*

3 *R* = (*C*_0_ − *C*_*t*_)/*C*_0_ × 100

where *C*_0_, *C*_*t*_, and *C*_e_ represent the initial, final, and equilibrium concentrations of atrazine in µg L^−1^, *V* is the solution volume (mL), and *M* is the amount of adsorbent (mg). The kinetics of the adsorption process were analyzed using pseudo-first and second-order models. In contrast, adsorption isotherms (Langmuir and Freundlich) were applied to determine the best model that describes the adsorption behavior of the adsorbent–adsorbate interaction.

## 3. Results and discussions

### 3.1. Surface morphology by scanning electron microscopy (SEM)

**Fig. 2:**
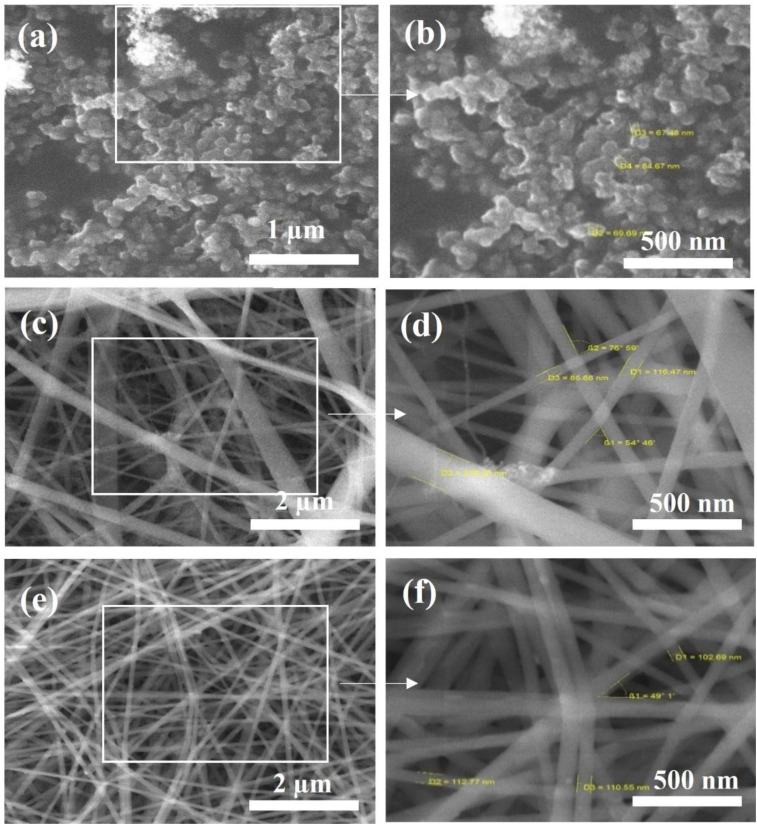
SEM images of (a and b) TiO_2_ NPs, (c and d) pristine nylon-6 nanofiber membrane, and (e and f) nylon-6/TiO_2_ nanofibrous membrane.

The SEM study was used to examine the surface morphology of TiO_2_ NPs (Fig. 2a and b), nylon-6 nanofibers (Fig. 2c and d), and TiO_2_-embedded nanofibers (Fig. 2e and f), respectively. The study revealed that as-synthesized TiO_2_ NPs exhibited regular shapes and were arranged orderly. It was also observed that the particles were spherically shaped, smooth, and uniformly distributed; the shape of the catalyst was an inhomogeneous structure; and no odd structures were detected on the surface. The size of TiO_2_ NPs was found to range from 100 to 300 nm, with an average particle size of 120–140 nm (Fig. S1). The small particle size provides a large surface area for hydroxyl ions (OH^−^) with the TiO_2_ surface to form hydroxyl radicals (OH˙), a potent oxidizing agent that oxidizes large amounts of pesticide molecules adsorbed on the surface of TiO_2_ NPs. Fig. 2c and d show the SEM images of the pristine nylon-6 nanofibrous membrane, and Fig. 2e and f shows TiO_2_ embedded composite fibers containing both micro- and nano-sized morphology of TiO_2_ NPs, revealing a rougher surface compared to the NPs multifilament yarns depicted. Most nanosized TiO_2_ NPs embedded in polymeric matrices have an average diameter of approximately 110 nm (Fig. S1). However, when TiO_2_ NPs were added, the fiber diameter of nylon-6/TiO_2_ increased by 10% from 100 nm to 110 nm. This study indicates that the intercalation of TiO_2_ NPs into the nylon-6 significantly changes its properties, and therefore, nanofibers with small diameters are formed. Such finer nanofibers enhance the number of fibers per unit area and increase the overall specific surface area. Therefore, due to these changes, the number of active sites for adsorption is increased. Consequently, nanofiber mats with smaller fiber diameters are expected to exhibit superior adsorption performance compared to those with larger diameters. However, some agglomeration of TiO_2_ NPs was observed on the fiber surfaces, attributed to interparticle attractive forces. Overall, both the fiber diameter and the size/distribution of NPs within the polymer matrix play a critical role in determining the final performance of the composite, as they directly influence dispersion uniformity, interfacial interactions, and bonding between the nanoparticles and the polymer chains.

### 3.2. Energy-dispersive spectroscopy (EDS) and Brunauer–Emmett–Teller (BET)

The linear EDS analysis was performed to investigate the distribution of nylon-6, TiO_2_ NPs and nylon-6/TiO_2_ nanofibrous membrane and element composition of the surface of nanofibers (Fig. S2a–c). These results confirmed the presence of titanium (24.33%), oxygen (46.23%), and carbon (29.44%) on the surface of nylon-6/TiO_2_ nanofibrous membrane (Fig. S2c), compared to pristine nylon-6 (Fig. S2a) and TiO_2_ NPs (Fig. S2b).

The BET surface areas of nylon-6/TiO_2_ and pristine nylon-6 membranes are shown in Fig. S3a and b, respectively. We observed that the specific surface area is increased from 10.12 m^2^ g^−1^ for the pristine nylon-6 membrane to 24.54 m^2^ g^−1^ for the nylon-6/TiO_2_ nanofibrous membrane, accompanied by a slight decrease in total pore volume (from 0.045 cm^3^ g^−1^ to 0.037 cm^3^ g^−1^), which may appear counter-intuitive at first glance, as shown in Fig. S3a, b and Table S1. This is due to the partial blockage of larger pores by TiO_2_ NPs coupled with the formation of new smaller mesopores and micropores at the polymer–NPs interfaces. The incorporation of TiO_2_ NPs into the nylon-6 nanofiber matrix leads to partial blockage of larger mesopores and macropores originally present in the pristine polymer nanofibers. This pore blockage reduces the overall total pore volume. However, the well-dispersed TiO_2_ NPs simultaneously introduce additional mesopores and micropores at the interfaces between the polymer chains and the inorganic NPs, as well as on the nanoparticle surfaces themselves. Because smaller pores (especially in the meso- and micro-range) contribute disproportionately more to the specific surface area (due to the inverse relationship between pore diameter and surface area), the net result is a significant increase in BET specific surface area despite the modest reduction in total pore volume. Furthermore, the TiO_2_ NPs can create surface roughness and additional interfacial area within the nanofiber structure, further enhancing the accessible surface for nitrogen adsorption during BET analysis. Fig. S3a shows the nitrogen adsorption–desorption isotherm, which exhibits a characteristic type IV shape with an H3/H4 hysteresis loop, typical of mesoporous materials, confirming the presence of mesopores in the composite. This trade-off decreased total pore volume with increased specific surface area is commonly reported in polymer–nanoparticle composite nanofibers and is generally beneficial for adsorption applications, as higher surface area provides more active sites for atrazine binding.

### 3.3. FTIR spectroscopy

**Fig. 3:**
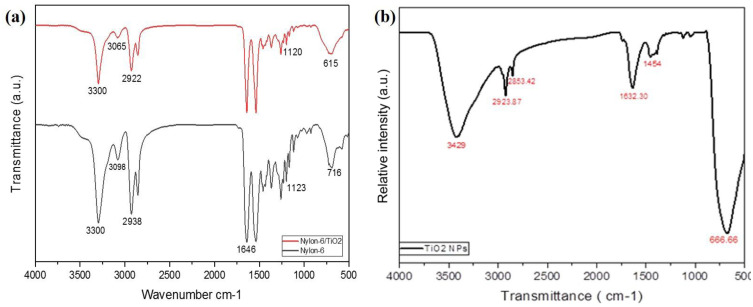
(a and b) FTIR spectra of pristine nylon-6, and nylon-6/TiO_2_ nanofibrous membranes. (b) FTIR spectrum of TiO_2_ NPs.

FTIR spectra of the as-synthesized TiO_2_ NPs and the nanofibrous membranes are shown in Fig. 3. The spectra provide insight into chemical bonding, surface hydroxyl groups, and possible interactions between TiO_2_ and nylon-6. The TiO_2_ NPs exhibited several characteristic peaks: <666.6 cm^−1^, broad Ti–O stretching vibrations, indicating the formation of TiO_2_ crystal networks,^25^ 1000–1200 cm^−1^, Ti–O–Ti stretching vibrations, confirming the interconnected TiO_2_ lattice structure, 1454 cm^−1^, C–F stretching and O–H bending vibrations, attributed to surface hydroxyl groups or adsorbed water, 1632 cm^−1^, C–H bending, likely from residual organic moieties from the precursor (TTIP), 2515–2923 cm^−1^, O–H and N–H stretching vibrations, indicating the presence of adsorbed moisture or hydroxyl groups on the nanoparticle surface, 3429–3450 cm^−1^, broad O–H stretching due to surface hydroxyl groups or physically adsorbed water. These peaks confirm the successful synthesis of TiO_2_ NPs with abundant surface hydroxyl groups, which are crucial for adsorption applications. The observed vibrations match well with reported data for anatase TiO_2_.

The FTIR spectrum of pristine nylon-6 nanofibers shows band at 3300 cm^−1^ which confirm the N–H stretching from amide groups, 3098 cm^−1^, secondary N–H stretching (Fermi resonance); 2938 cm^−1^, CH_2_ asymmetric stretching, 1646 cm^−1^, C=O stretching (amide I), 1543 cm^−1^, N–H bending and C–N stretching (amide II), 716 cm^−1^, CH_2_ in-plane bending. These characteristic bands confirmed the semi-crystalline structure of nylon-6 and the presence of hydrogen-bonded amide groups, consistent with previous reports.

While the spectrum of nylon-6/TiO_2_ nanofibrous membrane shows all the characteristic nylon-6 peaks with slight shifts, indicating interaction with TiO_2_ NPs, 2208 cm^−1^, a small red shift, suggesting weak interactions between the polymer chains and TiO_2_ NPs; 1463 cm^−1^: amide II (C=N stretching and CO–N–H bending), 615 cm^−1^, Ti–O stretching, confirming the presence and homogeneous distribution of TiO_2_ within the nanofibers. The main nylon-6 bands remain largely unchanged, indicating that the incorporation of TiO_2_ does not alter the polymer's primary chemical structure. The slight shifts and new Ti–O peak suggest physical embedding of TiO_2_ with possible weak hydrogen bonding or van der Waals interactions between nylon-6 and the NPs. These results suggested that the successful synthesis of anatase-phase TiO_2_ NPs with surface hydroxyl groups and semi-crystalline structure of nylon-6 nanofibrous membrane with characteristic amide I and II bands. While, weak polymer–nanoparticle interactions (physical embedding) that are sufficient to enhance surface reactivity for adsorption applications. These findings support the improved adsorption performance of nylon-6/TiO_2_ nanofibrous membranes observed in subsequent experiments.

### 3.4. X-ray diffraction (XRD) analysis

**Fig. 4:**
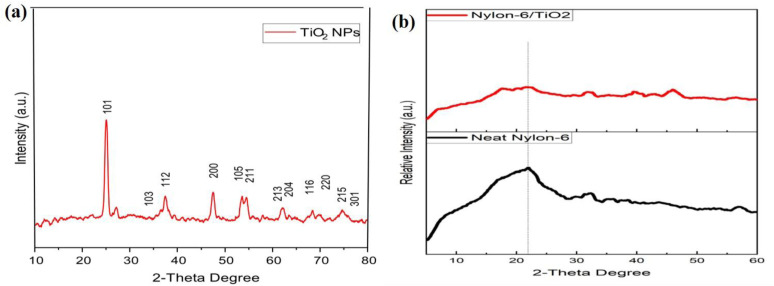
(a and b) XRD pattern of TiO_2_ NPs, pristine nylon-6 and nylon-6/TiO_2_ nanofibrous membranes, respectively.

XRD pattern was carried out for the identification of the TiO_2_ crystal phase and the structures of the micro- and nano-composite. Fig. 4a shows integrated patterns of TiO_2_ NPs with different TiO_2_ contents and exhibits the sharp diffraction peak at *hkl* values of (101) at 25.1°, (104) at 52.04°, (112), (200) at 48.72°, (211) at 55.10°, (204) at 62.79°, (116) at 68.76°, (220) at 70.31°, (215) at 75.01° and (301) at 78.03°. All the peaks are in good arrangement with JCPDS card number 21-1272 (anatase TiO_2_), and the XRD pattern is reported.^26^ The prominent peak at approximately 25.1° corresponds to the (101) crystal plane, which is the signature peak for anatase. This XRD pattern confirms that as-synthesized NPs have pure anatase TiO_2_. The sample appears to be phase-pure (no visible impurities or secondary phases) and possesses a well-ordered crystalline structure at the nanometer scale and are in strong agreement with previously published data.^27^ A minor rutile phase appeared at higher calcination temperatures (above 700 °C), indicating partial phase transformation. A characteristic peak designated at 21.8° exhibits a detrimental height due to the plasticization effect of the polymer that promoted the formation of semi-crystalline nylon-6.

The nylon-6 nanofibrous membranes (Fig. 4b) show broad peaks at 20.6° and 21.5°, corresponding to the (200) and (200/002) planes of nylon-6. These broad features are typical of semi-crystalline polymers. Fig. 4b exhibits diffraction peaks of the composite of nylon-6/TiO_2_ that are still clearly observed, particularly the prominent one at 22.4° at the (101) peak. The retention of the anatase peak in the composite confirms that the crystalline phase of TiO_2_ is preserved during processing. There is no significant shift in peak positions, although there is a slight broadening and intensity reduction of the sharp peak of TiO_2_ along the fiber contour, likely due to the interaction and possible surface adsorption of TiO_2_ NPs.

### 3.5. Tensile testing

The tensile strength of nylon-6 and nylon-6/TiO_2_ was calculated using a universal tensile testing machine (Titan Universal Tester 3-910 Company Ltd, Germany) under the ASTM D-2256 method. The crosshead speed on the universal testing machine was set at 1.0 mm min^−1^. The thickness of nylon-6/TiO_2_ nanofibrous membrane increases 21 µm to 39 µm. The thickness was improved with the incorporation of TiO_2_ NPs in the solution. The incorporation of TiO_2_ significantly enhanced the mechanical performance of the membranes. The as-prepared nylon-6/TiO_2_ nanofibrous membrane showed with average tensile strength ∼ 1.2 MPa, which is slightly higher than pristine nylon-6 membranes (∼1.0 MPa). The slight increase in tensile strength is attributed to hydrogen bonding between the amide groups of nylon-6 and the surface hydroxyl groups of TiO_2_, which enhances fiber cohesion and mechanical stability.

## 4. Adsorption studies

### 4.1. Effect of pH and concentration variation on the adsorption of atrazine

**Fig. 5:**
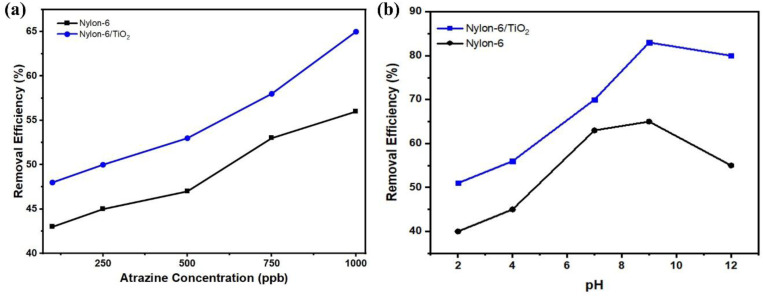
(a) Removal percentage at different atrazine concentration, and (b) effect of pH solution on atrazine adsorption.

The influence of adsorbent dosage was studied by varying the nanofibrous membrane mass from 2 to 10 mg while maintaining other parameters constant. Fig. 5a shows that increasing the adsorbent dosage enhanced atrazine removal for both membranes due to the increased availability of active adsorption sites. Atrazine removal increased from 45.1% to 54% for pristine nylon-6 and from 57% to 67% for nylon-6/TiO_2_ nanofibrous membranes. The effect of contact time was evaluated between 30 and 150 minutes at an atrazine concentration of 1000 ppb. Fig. 5b exhibits that the adsorption increased rapidly during the initial period and gradually approached equilibrium. Maximum removal efficiencies of 68% (nylon-6) and 85.67% (nylon-6/TiO_2_) were achieved at 30 minutes, beyond which no significant improvement was observed, indicating equilibrium. The enhance performance of nylon-6/TiO_2_ nanofibrous membranes is attributed to increased surface area and stronger physicochemical interactions with atrazine molecules.

### 4.2. Effect of adsorbent dosage and time variation on the adsorption of atrazine

**Fig. 6:**
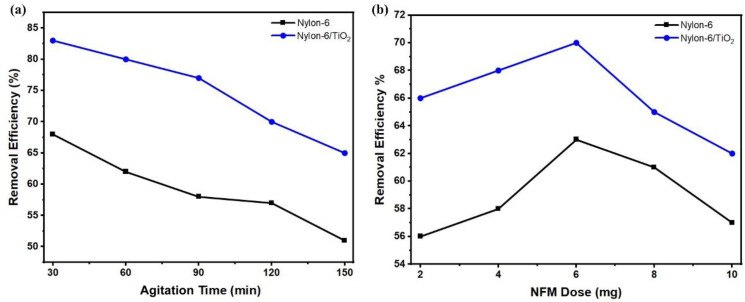
(a) Effect of contact time on atrazine adsorption. (b) Effect the dosage of adsorbent on removal percentage of atrazine adsorption (1000 µg L^−1^).

The influence of adsorbent dosage was studied by varying the nanofibrous membrane mass from 2 to 10 mg while maintaining other parameters constant. Fig. 6a shows that increasing the adsorbent dosage enhanced atrazine removal for both membranes due to the increased availability of active adsorption sites. Atrazine removal increased from 45.1% to 54% for pristine nylon-6 and from 57% to 67% for nylon-6/TiO_2_ nanofibrous membranes. The effect of contact time was evaluated between 30 and 150 minutes at an atrazine concentration of 1000 µg L^−1^. Fig. 6b exhibits adsorption increasing rapidly during the initial period and gradually approaching equilibrium. Maximum removal efficiencies of 68% (nylon-6) and 85.67% (nylon-6/TiO_2_) were achieved at 30 min, beyond which no significant improvement was observed, indicating equilibrium. The enhanced performance of nylon-6/TiO_2_ nanofibrous membranes is attributed to increased surface area and stronger physicochemical interactions with atrazine molecules.

### 4.3. Liquid chromatography/mass spectrometry (LC-MS/MS)

**Fig. 7:**
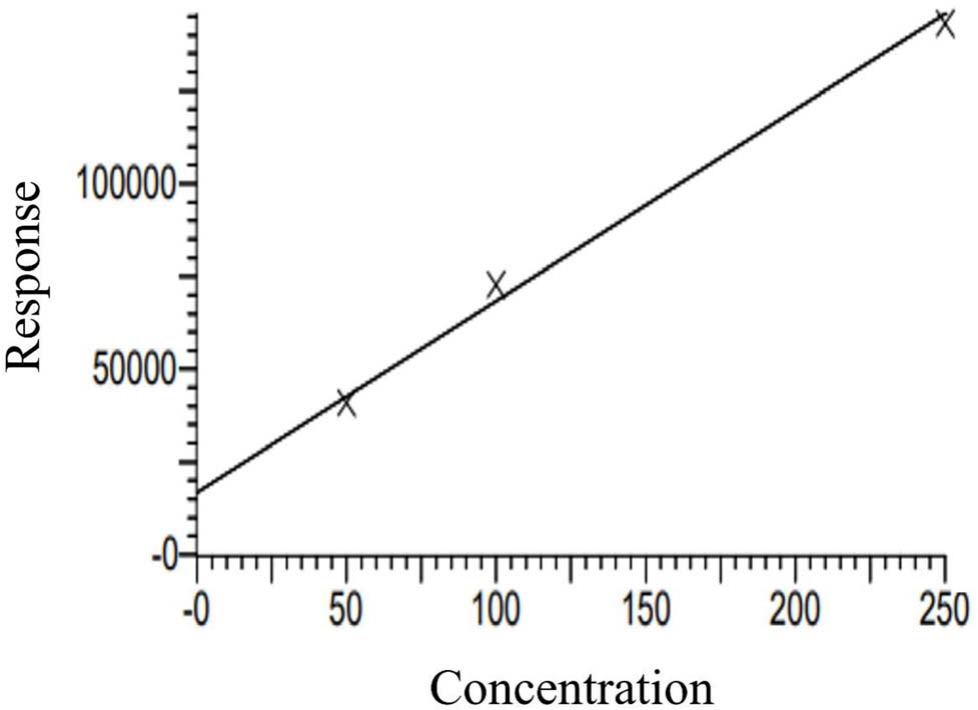
Calibration curve data for atrazine standards and blanks before sample analysis in liquid chromatography mass–mass spectrometry.

The quantitative analysis of atrazine was performed using the LC-MS/MS. The calibration curve for atrazine, with a correlation coefficient (*R* = 0.996277) and a coefficient of determination (*R*^2^ = 0.992), the atrazine calibration curve exhibits exceptional linearity and dependability for measuring the analyte (Fig. 7). The sensitivity of the method is indicated by the calibration curve's slope value (*m*): 515.9, which represents the rate at which the response varies with concentration *x*. Greater sensitivity is indicated by a steeper slope, which means that even slight changes in concentration cause a significant increase in the response, such as area and the intercept value of 16 777.2, which is probably the result of instrumental background noise or other systematic factors, represents the baseline signal or offset. Three standards and two blanks served as the basis for the calibration, which produced a clear and reliable curve. Among the three calibration standards were a 50 µg L^−1^ concentration with an accuracy of 93.7%, 100 µg L^−1^ with an accuracy of 108%, and 250 µg L^−1^ with an accuracy of 97.9%. The limit of quantification (LOQ), which represents the lowest concentration that can be precisely and accurately measured, was approximately 9.7 µg L^−1^, while the limit of detection (LOD), which represents the lowest detectable concentration, was estimated to be around 3.2 µg L^−1^ using an estimated standard deviation (SD = 500). The weighting factor guarantees increased precision at trace concentration levels, while the high accuracy across the three standards validates the method's dependability.

**Fig. 8:**
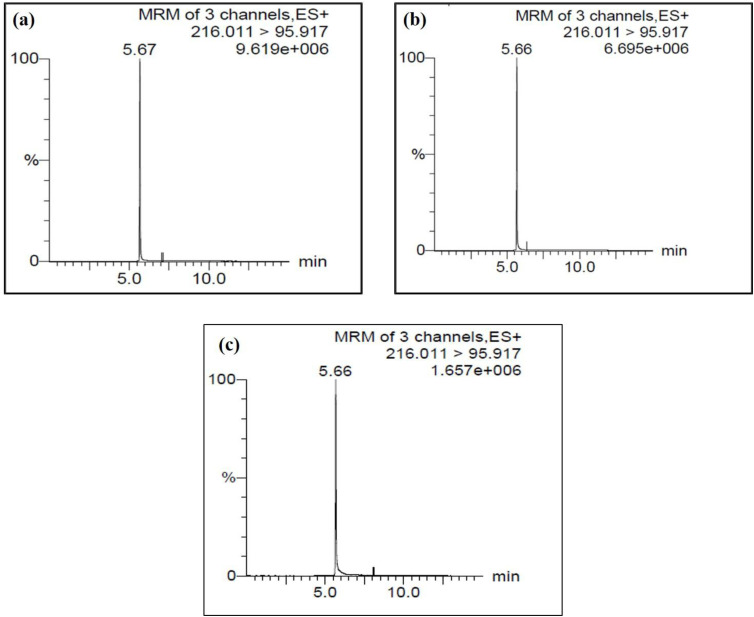
(a–c) LC-MS/MS chromatograms of synthetic water (1000 ppb), before (a), after (b) treatment with nylon-6, and (c) after treatment with nylon-6/TiO_2_.

Atrazine was monitored in the ESI^+^ mode using an MRM transition from the precursor ion at *m*/*z* 216.01 to the product ion at *m*/*z* 95.917. At an initial atrazine concentration of 1000 ppb, a signal intensity of 9.619 × 10^6^ was observed, indicating a strong and reliable analyte response (Fig. 8a–c). Based on the calibration curve, the measured concentration of atrazine in the sample was close to the initial value, allowing accurate determination of removal efficiency. Fig. 8 shows that the signal intensities decreased to 6.695 × 10^6^ and 1.657 × 10^6^ after treatment, respectively, demonstrating a substantial reduction in atrazine concentration. This decrease in signal intensity confirms effective adsorption of atrazine. Furthermore, the calculated removal efficiency was approximately 85%, as illustrated in Fig. 8.

### 4.4. Adsorption kinetics studies

**Fig. 9:**
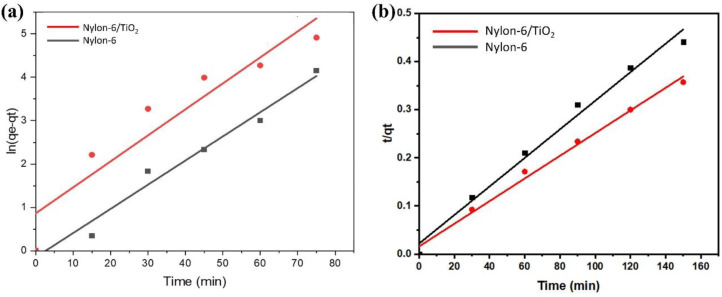
(a) Pseudo-first order and (b) pseudo-second order kinetic models.

Kinetic studies were carried out to comprehend the adsorption mechanism between the adsorbed and adsorbate (Fig. 9a and b). The adsorption efficiency of the adsorbent is determined by the kinetics of the adsorbent adsorption rate. Therefore, the experimental data was presented by the kinetic model pseudo first-order (Fig. 9a) and pseudo second-order (Fig. 9b) to describe the transfer of mass process. Using pseudo first- and second-order kinetics models, researchers investigated the adsorption process mechanism and calculated the adsorption capacity and rate constant of atrazine. Eqn (4) and (5) illustrate the pseudo differential form of kinetic equations that are first-order and pseudo second-order respectively.4
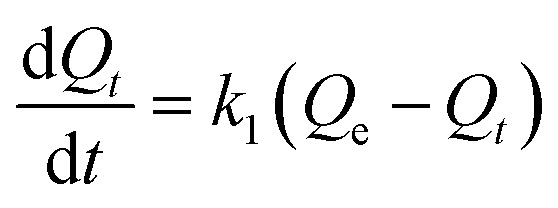
5
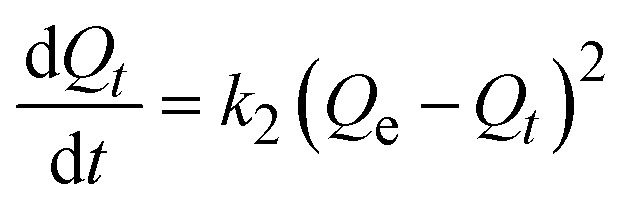
Here, *Q*_e_ and *Q*_*t*_ are the adsorption capacity (µg g^−1^) at equilibrium and time (minutes), respectively; *k*_1_ and *k*_2_ are the pseudo first order (min^−1^) and pseudo second order (g µg^−1^ min^−1^), rate constants, respectively. The linear forms of eqn (4) and (5) are expressed as eqn (6) and (7) below respectively.6
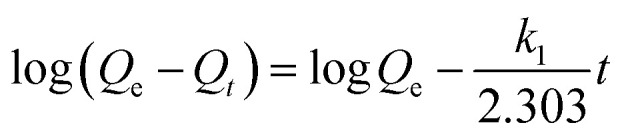
7
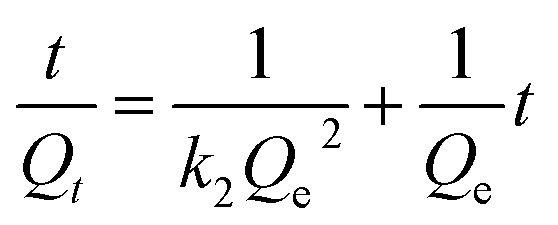


The kinetics parameters are summarized in Table 1. The data utilized to determine the adsorption kinetics came from examining the information in Section 4.1, *i.e.*, effect of contact time. The adsorption of atrazine has been found to follow pseudo-second-order kinetics. Uptake by pristine nylon-6 or nylon-6/TiO_2_ nanofibrous membranes is demonstrated by the greater proximity between experimental and theoretical adsorption efficiency and correlation (*R*^2^) values as compared to those of pseudo-first-order. According to the pseudo second-order best fit, chemisorption is the adsorption mechanism greater than physisorption. Due to its abundance of functional groups that promote chemisorption, the nylon-6/TiO_2_ supra molecule is favourable to this type of interaction. The plots' intercept can be used to assess the boundary layer effect for the adsorption. In other words, when surface adsorption makes a larger contribution towards plot intercepts will not equal to zero if the rate-controlling step occurs before the interparticle diffusion. In this study, the pseudo 2nd order is considered as best fit that means adsorption process followed chemisorption mechanism. Overall, the pseudo-second-order kinetic model was identified as the best fit, confirming that the adsorption process is predominantly governed by chemisorption (Fig. 9b). The nylon-6/TiO_2_ nanofibrous membranes exhibit high adsorption capacity and the adsorption capacities of atrazine on nylon-6/TiO_2_ nanofibrous membranes in comparison with other reported adsorbents are shown in Table 4.

**Table 1:** Adsorption kinetic model rate constants and parameters for atrazine adsorption on nylon-6 and nylon-6/TiO_2_ nanofibrous membranes

Adsorption kinetics						
Adsorbent	Pseudo 1st order kinetics			Pseudo 2nd order kinetics		
	*k*_1_	*Q*_e_ (mg g^−1^)	*R*_1_^2^	*k*_2_	*Q*_e_ (mg g^−1^)	*R*_2_^2^
Nylon-6/TiO_2_	0.00185	21.1189	0.971	0.00346	42.309	0.99648
Nylon-6	0.001998	22.471	0.834	0.0041	33.700	0.9906

### 4.5. Isotherm studies

Isotherm studies are essential for understanding the adsorption behaviour of an adsorbent and for optimizing the design of the adsorption process. In this study, the experimental data were analysed using Langmuir and Freundlich adsorption isotherm models.^28^ The slope and intercept obtained from the linear forms of these models were used to calculate the corresponding isotherm parameters. The Langmuir isotherm model assumes that adsorption occurs as a monolayer on a homogeneous surface with a finite number of identical adsorption sites.^29^ As compared to the Langmuir model, the Freundlich isotherm model demonstrates the multilayer adsorption on a heterogeneous surface with sites of varying adsorption energies. These isotherm adsorptions provide important insights into the underlying adsorption mechanism, overall system behavior, and the specific surface properties of the adsorbent. The linearized forms of the Langmuir and Freundlich isotherm equations were utilized (Table 2).

**Table 2:** Langmuir and Freundlich linear models for atrazine adsorption on nylon-6 and nylon-6/TiO_2_ membranes^a^

Model	Linear
Langmuir	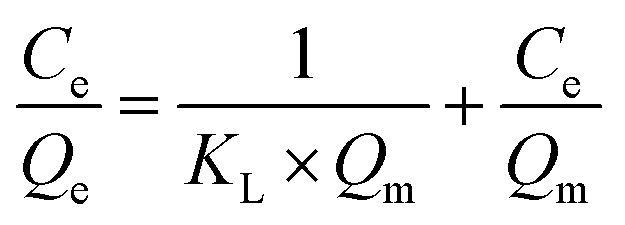
Freundlich	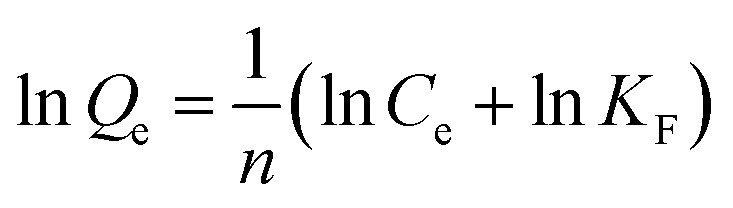

a *Q*_e_ is the adsorption capacity at equilibrium (µg g^−1^), *C*_e_ is the concentration at equilibrium (mg L^−1^), *K*_F_ and *Q*_m_ are the constant of Langmuir associated with the binding energy and maximum adsorption capacity, and an is the heterogeneity factor.

### 4.6. Adsorption of pollutants by nylon-6/TiO_2_ nanofibrous membrane

**Fig. 10:**
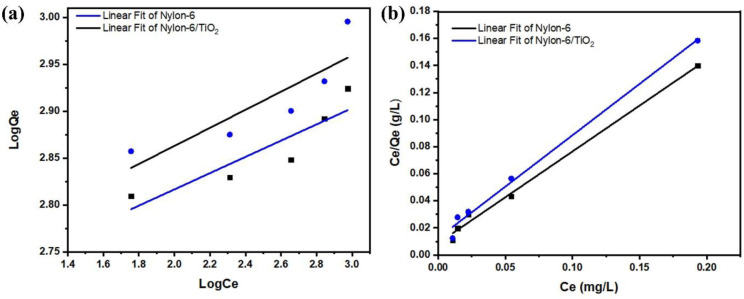
(a) Freundlich and (b) Langmuir isotherms models.

The nylon-6/TiO_2_ nanofibrous membrane showed a high absorption capability for absorption of various pollutants due to its different interaction including van der Waals forces and molecular entrapment within its structure. The maximum adsorption capacities were determined to be 52.79 mg g^−1^ for nylon-6 and 67.10 mg g^−1^ for nylon-6/TiO_2_, demonstrating the improved adsorption performance resulting from TiO_2_ incorporation. The Freundlich (Fig. 10a) and Langmuir (Fig. 10b) isotherm parameters for both nylon-6 and nylon-6/TiO_2_ are shown in Table 3. Isotherm analysis indicated that the Langmuir model provided the best fit for the experimental data of the synthesized nanofibrous membranes, as illustrated in Fig. 10. These results are well matched with values reported in literature. Then, the obtained experimental results using varying initial concentrations of atrazine were validated by performing linear curve fitting of the Langmuir isotherm. The decrease in concentration of atrazine after adsorption experiments was studied after optimization of conditions, which can be attributed to the availability of increased active adsorption sites on the membrane surface (Fig. S4). The results provide strong evidence for the formation of a monolayer of atrazine molecules on the surface of the nylon-6/TiO_2_ nanofibrous membrane. Furthermore, the good agreement with the Langmuir model confirms that chemisorption is the dominant mechanism governing the uptake of atrazine from the aqueous solution. Further, as-obtained results are several folds greater than reported works in literature, as shown in Table 4.

**Table 3:** Adsorption isotherms Langmuir and Freundlich linear model rate constants and parameters for atrazine adsorption on nylon-6 and nylon-6/TiO_2_ nanofibrous membranes

Adsorption isotherms study						
Membrane	Langmuir			Freundlich		
	*b* (L mg^−1^)	*Q*_max_ (mg g^−1^)	*R*_1_^2^	*k*_2_	*n*	*R*_2_^2^
Nylon-6/TiO_2_	0.00185	67.12	0.9974	0.00346	42.309	0.759
Nylon-6	0.001998	52.79	0.9903	0.0041	33.700	0.667

**Table 4:** Comparison of this study with other adsorbent materials in literature

Type of adsorbent	Adsorbent dose	Maximum adsorption capacity	Removal (%)	Ref.
PAN–carbon/TiO_2_	30 mg	—	40–90	30
PAN nanofibers	0.336 g	10.08 mg g^−1^	76	31
PA6/PPY NFM	2.0 mg	14.8 mg g^−1^	74	32
Multiwalled carbon nanotubes		40.16 mg g^−1^	70	33
Nanoscale zero valent iron	200 mg	8.89 mg g^−1^	73	34
Powdered activated carbon (PAC, commercial)	18.50	—	2–35%	35
Graphene oxide (GO)	1011.94	—	>80–98% (in cycles)	36
Banana stem AC (BSAC)		13.95–712.10 mg g^−1^	85.36%	37
PVP-*co*-S–clay composite	0.367% w/w	—	90–99%	38
H_2_O_2_-modified PAC	18.5 mg	—	80% (1 h)	39
**Pure nylon-6 membrane**	**6.0 mg**	**51.79 mg g^−^** ^**1**^	**∼69%**	**Current study**
**Nylon-6/TiO**_**2**_ **nanofiber membrane**	**6.0 mg**	**67.12 mg g^−^** ^**1**^	**85.6**	**Current study**

## 5. Conclusion

This study demonstrated the simple and cost-effective modification strategy for the adsorption of atrazine by applying nylon-6/TiO_2_ nanofibrous membranes. The intercalation of TiO_2_ NPs into the nylon-6 matrix was successfully confirmed by FTIR, XRD, BET, and SEM techniques. XRD patterns verified the presence of TiO_2_ within the polymer matrix, while FTIR spectra revealed strong interactions between TiO_2_ NPs and the amide functional groups of nylon-6. SEM images showed a uniform and continuous nanofibrous structure with average fiber diameters in the range of 110–130 nm and membrane thicknesses between 21 µm and 39 µm. The as-prepared nylon-6/TiO_2_ nanofibrous membrane showed with average tensile strength ∼ 1.2 MPa, which is slightly higher than pristine nylon-6 membranes (∼1.0 MPa). Adsorption studies describes that the Langmuir isotherm model provided the best fit to the experimental results, yielding a high correlation coefficient (*R*^2^ = 0.997). In addition, the adsorption kinetics followed a pseudo-second-order model, suggesting the involvement of both physisorption and chemisorption mechanisms. As a result, the maximum adsorption capacity increased from 52.79 mg g^−1^ for pristine nylon-6 membranes to 67.12 mg g^−1^ for nylon-6/TiO_2_ nanofibrous membranes.

## Conflicts of interest

The authors declare that there is no conflict of interest for this work.

## Supplementary Material

RA-016-D6RA02398C-s001

## Data Availability

Data supporting the finding of this work are available in this article and its supplementary information (SI). Additional datasets used and/or analyzed in this study are available from the corresponding authors upon request. Supplementary information is available. See DOI: https://doi.org/10.1039/d6ra02398c.

## References

[cit1] Hyder A., Khilji M.-U.-N., Buledi J. A., Memon A. A., Ghanghro A., ur Rehman M., Thebo K. H. (2025). MXene-based nanocomposites: a new horizon for electrochemical monitoring of environmental pollutants. RSC Sustainability.

[cit2] Mehdi M., Jiang W., Zeng Q., Thebo K. H., Kim I.-S., Khatri Z., Wang H., Hu J., Zhang K.-Q. (2022). Regenerated Silk Nanofibers for Robust and Cyclic Adsorption–Desorption on Anionic Dyes. Langmuir.

[cit3] Tariq M. I., Afzal S., Hussain I., Sultana N. (2007). Pesticides exposure in Pakistan: A review. Environ. Int..

[cit4] Tahir R., Afzal F., Jamil H., Razzaq M., Khan M. S. (2024). Physiological impacts of pesticidal contamination: Challenges to sustainable agriculture and biodegradation methods. Adv. Agric. Anim. Sci..

[cit5] Lebelo K., Malebo N., Mochane M. J. (2021). Chemical Contamination Pathways and the Food Safety Implications along the Various Stages of Food Production: A Review. Int. J. Environ. Res. Public Health.

[cit6] Eddleston M. (2024). Poisoning by pesticides. Medicine.

[cit7] Rath P. C. , Gowda B., Pradhana S. S., Adak T., Guru-Pirasanna-Pandi G., Patil N. B. and Annamalai M., Pesticides Occurrence in Water Sources and Decontamination Techniques, in Pesticides – Updates on Toxicity, Efficacy and Risk Assessment, ed. M. L. Larramendy and S. Soloneski, IntechOpen, London, 2022

[cit8] Shekhar C., Khosya R., Thakur K., Mahajan D., Kumar R., Kumar S., Sharma A. K. (2024). A systematic review of pesticide exposure, associated risks, and long-term human health impacts. Toxicol. Rep..

[cit9] Steliarova-Foucher E., Colombet M., Ries L. A. G., Moreno F., Dolya A., Bray F., Hesseling P., Shin H. Y., Stiller C. A. (2017). International incidence of childhood cancer, 2001-10: a population-based registry study. Lancet Oncol..

[cit10] Teklu B. M., Adriaanse P. I., Ter Horst M. M. S., Deneer J. W., Van den Brink P. J. (2015). Surface water risk assessment of pesticides in Ethiopia. Sci. Total Environ..

[cit11] Rostami S., Jafari S., Moeini Z., Jaskulak M., Keshtgar L., Badeenezhad A., Azhdarpoor A., Rostami M., Zorena K., Dehghani M. (2021). Current methods and technologies for degradation of atrazine in contaminated soil and water: A review. Environ. Technol. Innovation.

[cit12] Kuchenbaecker K. B., Hopper J. L., Barnes D. R., Phillips K.-A., Mooij T. M., Roos-Blom M.-J., Jervis S., van Leeuwen F. E., Milne R. L., Andrieu N., Goldgar D. E., Terry M. B., Rookus M. A., Easton D. F., Antoniou A. C., BRCA1 and BRCA2 Cohort Consortium (2017). Risks of Breast, Ovarian, and Contralateral Breast Cancer for BRCA1 and BRCA2 Mutation Carriers. JAMA, J. Am. Med. Assoc..

[cit13] Memon F. H., Rehman F., Lee J., Soomro F., Iqbal M., Khan S. M., Ali A., Thebo K. H., Choi K. H. (2023). Transition Metal Dichalcogenide-based Membranes for Water Desalination, Gas Separation, and Energy Storage. Sep. Purif. Rev..

[cit14] Tanveer R., Yasar A., Nizami A.-S., Tabinda A. B. (2023). Integration of physical and advanced oxidation
processes for treatment and reuse of textile dye-bath effluents with minimum area footprint. J. Cleaner Prod..

[cit15] Yong Z. Y., Othman M. H. D., Yong E. L., Puteh M. H., Jaafar J., Rahman M. A., Kurniawan T. A. (2026). Global Occurrence, Health Risks, and Treatment Challenges of PFAS in Wastewater: Prospects for Photocatalytic Membrane Technologies. Int. J. Environ. Res..

[cit16] Li J., Cheng W., Wang H., Luo Y., Liu Q., Wang X., Wang L., Zhang T. (2025). Reverse osmosis and nanofiltration processes in industrial wastewater treatment: The recent progress, challenge, and future opportunity. Sep. Purif. Technol..

[cit17] Hassaan M. A., El-Nemr M. A., Elkatory M. R., Ragab S., Niculescu V. C., El Nemr A. (2023). Principles of Photocatalysts and Their Different Applications: A Review. Top. Curr. Chem..

[cit18] Satyam S., Patra S. (2024). Innovations and challenges in adsorption-based wastewater remediation: A comprehensive review. Heliyon.

[cit19] Shahzad M. K., Memon F. H., Soomro F., Iqbal M., Ibrar A., Memon A. A., Lim J. H., Choi K. H., Thebo K. H. (2023). MoS_2_-based lamellar membranes for mass transport applications: Challenges and opportunities. J. Environ. Chem. Eng..

[cit20] Gavrilaş S. (2025). Nanomembranes as Eco-Friendly Instruments for Modern Food Processing, from Filtration to Packaging. Membranes.

[cit21] Janjhi F. A., Janwery D., Chandio I., Ullah S., Rehman F., Memon A. A., Hakami J., Khan F., Boczkaj G., Thebo K. H. (2022). Recent Advances in Graphene Oxide-Based Membranes for Heavy Metal Ions Separation. ChemBioEng Rev..

[cit22] He X., Guo Y., Liu J., Li X., Qi J. (2019). Fabrication of peanut-like TiO_2_ microarchitecture with enhanced surface light trapping and high specific surface area for high-efficiency dye sensitized solar cells. J. Power Sources.

[cit23] Ali Z., Mehmood M., Ahmed J., Majeed A., Thebo K. H. (2020). CVD grown defect rich-MWCNTs with anchored CoFe alloy nanoparticles for OER activity. Mater. Lett..

[cit24] Ahmed Z., Rehman F., Ali U., Ali A., Iqbal M., Thebo K. H. (2021). Recent Advances in MXene-based Separation Membranes. ChemBioEng Rev..

[cit25] Rab N., Chong F. K., Mohamed H. I., Lim W. H. (2018). Preparation of TiO_2_ nanoparticles by hydrolysis of TiCl_4_ using water and glycerol solvent system. J. Phys.: Conf. Ser..

[cit26] Nagaraj G., Brundha D., Chandraleka C., Arulpriya M., Kowsalya V., Sangavi S., Jayalakshmi R., Tamilarasu S., Murugan R. (2020). Facile synthesis of improved anatase TiO_2_ nanoparticles for enhanced solar-light driven photocatalyst. SN Appl. Sci..

[cit27] Sellami H., Akinyemi M. O., Gdoura-Ben Amor M., Onwudiwe D. C., Mthiyane D. M. N. (2025). Structural and optical characterization of TiO_2_ nanoparticles synthesized using Globularia alypum leaf extract and the antibacterial properties. Discover Appl. Sci..

[cit28] Khayyun T. S., Mseer A. H. (2019). Comparison of the experimental results with the Langmuir and Freundlich models for copper removal on limestone adsorbent. Appl. Water Sci..

[cit29] Kanwal S., Devi P., Ahmed Z., Qambrani N. A. (2024). Adsorption isotherm, kinetic and thermodynamic studies for adsorption of fluoride on waste marble powder. Desalin. Water Treat..

[cit30] Greenstein K. E., Nagorzanski M. R., Kelsay B., Verdugo E. M., Myung N. V., Parkin G. F., Cwiertny D. M. (2021). Carbon–titanium dioxide (C/TiO_2_) nanofiber composites for chemical oxidation of emerging organic contaminants in reactive filtration applications. Environ. Sci.: Nano.

[cit31] Chabalala M. B., Gumbi N. N., Mamba B. B., Al-Abri M. Z., Nxumalo E. N. (2021). Photocatalytic Nanofiber Membranes for the Degradation of Micropollutants and Their Antimicrobial Activity: Recent Advances and Future Prospects. Membranes.

[cit32] Yang B.-Y., Cao Y., Qi F.-F., Li X.-Q., Xu Q. (2015). Atrazine adsorption removal with nylon6/polypyrrole core-shell nanofibers mat: possible mechanism and characteristics. Nanoscale Res. Lett..

[cit33] Tang W.-W., Zeng G.-M., Gong J.-L., Liu Y., Wang X.-Y., Liu Y.-Y., Liu Z.-F., Chen L., Zhang X.-R., Tu D.-Z. (2012). Simultaneous adsorption of atrazine and Cu(II) from wastewater by magnetic multi-walled carbon nanotube. Chem. Eng. J..

[cit34] Zhang Y., Li Y., Zheng X. (2011). Removal of atrazine by nanoscale zero valent iron supported on organobentonite. Sci. Total Environ..

[cit35] Demba N’diaye A. (2022). Removal of atrazine from aqueous solution onto commercial Activated Carbons. J. Mater. Environ. Sci..

[cit36] Cheng C., Shi X., Yin G., Peng F., Hou W., Zhang W., Lin X., Li J., Wang X. (2022). Atrazine adsorption by graphene-based materials: Interaction mechanism and application in real samples. Environ. Technol. Innovation.

[cit37] Amidu H., Kiti J., Annan E., Arkorful G. K., Owusu Asimeng B., Aba Modupeh Hodasi J. (2025). Mitigation of atrazine pesticide using banana stem-derived activated carbon. Environ. Sci. Pollut. Res..

[cit38] Zadaka D., Nir S., Radian A., Mishael Y. G. (2009). Atrazine removal from water by polycation–clay composites: Effect of dissolved organic matter and comparison to activated carbon. Water Res..

[cit39] Callao C., Mallari M. C., Mangubat M., Haranay H. (2026). Atrazine Removal from Aqueous Solutions Using Hydrogen Peroxide-Modified Powdered Activated Carbon. Int. J. Transdiscipl. Res. Perspect..

