# Supplementary material for: Highly efficient TiO2-functionalized nylon-6 nanofibrous membranes for rapid adsorptive removal of atrazine from water
Source: RSC Adv. 2026 May 21;16(30):27481–94. doi: 10.1039/d6ra02398c (PMC13195425; doi:10.1039/d6ra02398c)
Supplement: RA-016-D6RA02398C-s001 [file RA-016-D6RA02398C-s001.pdf]

Supplementary Information (SI) for RSC Advances.
This journal is © The Royal Society of Chemistry 2026

Supporting Data

Highly Efficient TiO_2-Functionalized Nylon-6 Nanofibrous Membranes for Rapid Adsorptive Removal of Atrazine from Water

Saira Sidhu[1], Syeda Sara Hassan[1]*, Muhammad Rizwan[1], Zeeshan Khatri[2], Safina Kamboh [1], Akbar Ali[3], Khalid Hussain Thebo[4*], Ahmed Nadeem[5]

[1]U.S - Pakistan Centre for Advanced Studies in Water, Mehran University of Engineering & Technology, Jamshoro, Pakistan

[2]Department of Textile Engineering, Mehran University of Engineering & Technology, Jamshoro, Pakistan

[3]MIIT Key Laboratory of Critical Materials Technology for New Energy Conversion and Storage, State Key Laboratory of Urban Water Resource and Environment, School of Chemistry and Chemical Engineering, Harbin Institute of Technology, Harbin 150001, PR China

[4]Department of Chemistry, Mirpur University of Science & Technology (MUST), Mirpur, A&J Kashmir, Pakistan

[5]Department of Pharmacology and Toxicology, College of Pharmacy, King Saud University, Riyadh 11451, Saudi Arabia

*Corresponding Authors: sshassan.uspcasw@faculty.muet.edu.pk (S.S.H.)**

*khalidthebo@yahoo.com (K.H.T.)**

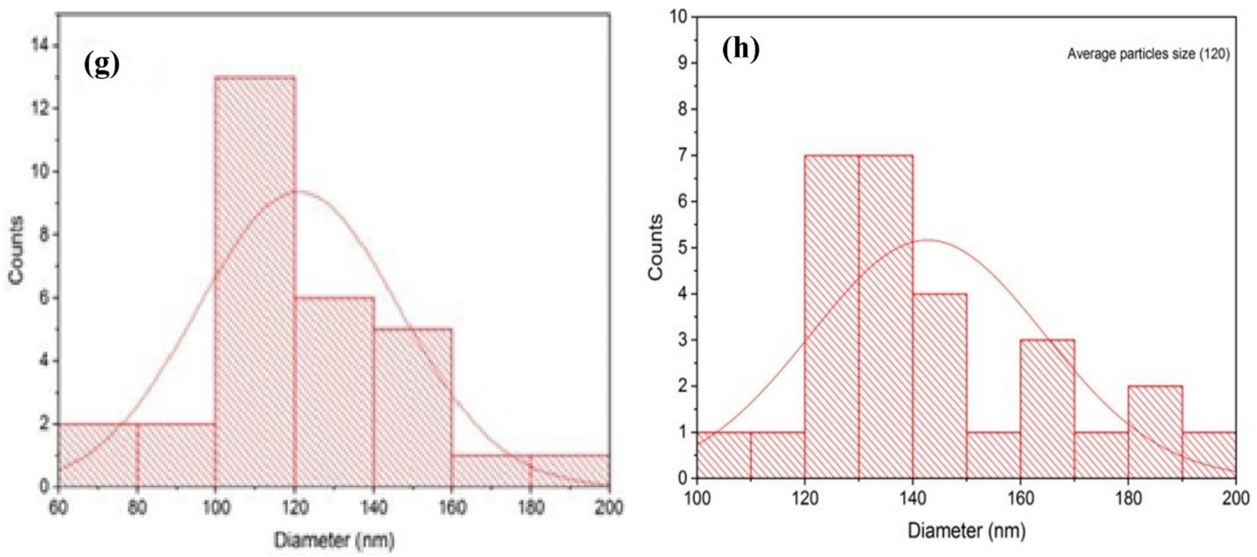

Fig. S1. Histogram showing average particle size of TiO_2 nanoparticles and nanofibers.

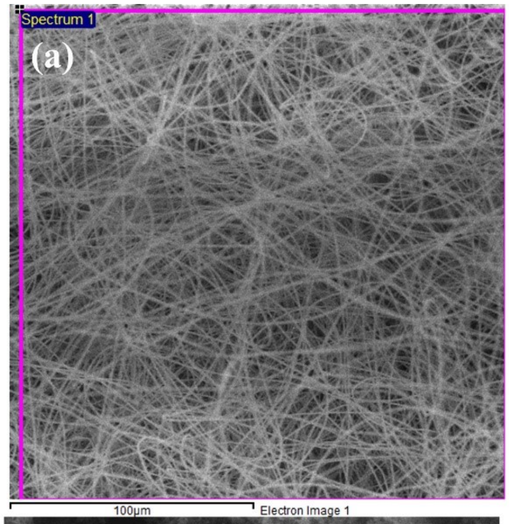

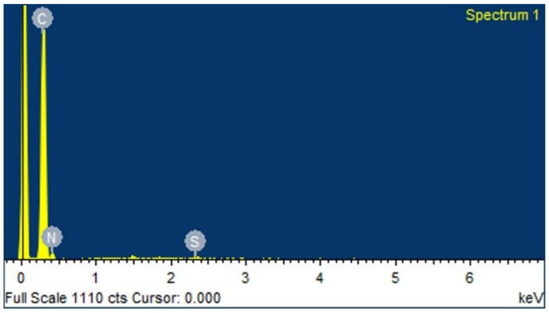

Element	Weight%	Atomic%
C K	83.58	85.99
N K	12.98	11.45
O K	3.18	2.46
S K	0.27	0.10

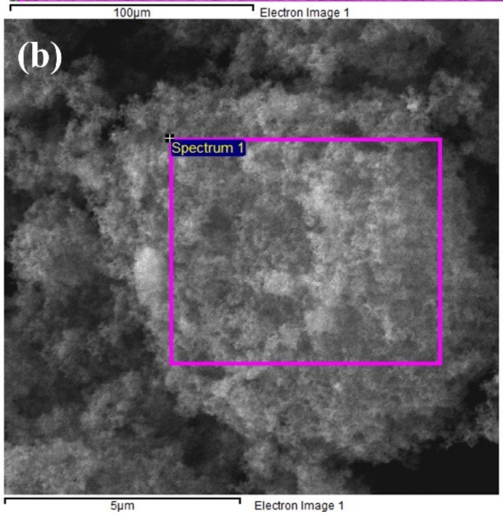

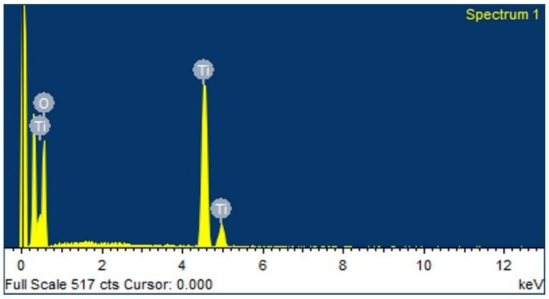

Element	Weight%	Atomic%
O K	64.16	84.28
Ti K	35.84	15.72
Totals	100.00	

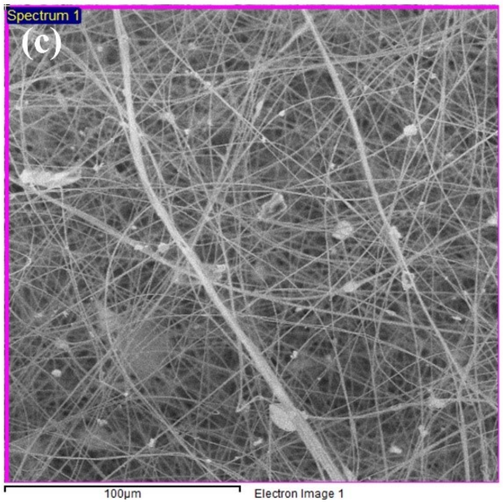

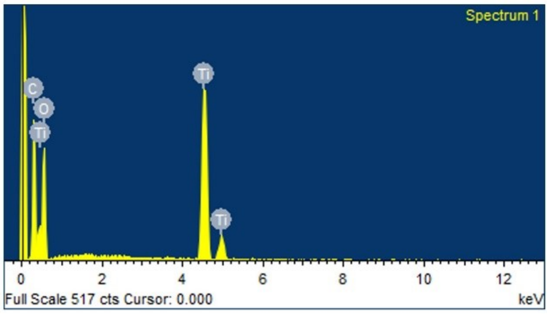

Element	Weight%	Atomic%
C K	29.44	41.91
O K	46.23	49.41
Ti K	24.33	8.69
Totals	100.00	

Fig. S2. EDS studies of (a) nylon-6, (b) TiO_2 NPs and (c) nylon-6/TiO_2 nanofibrous membranes

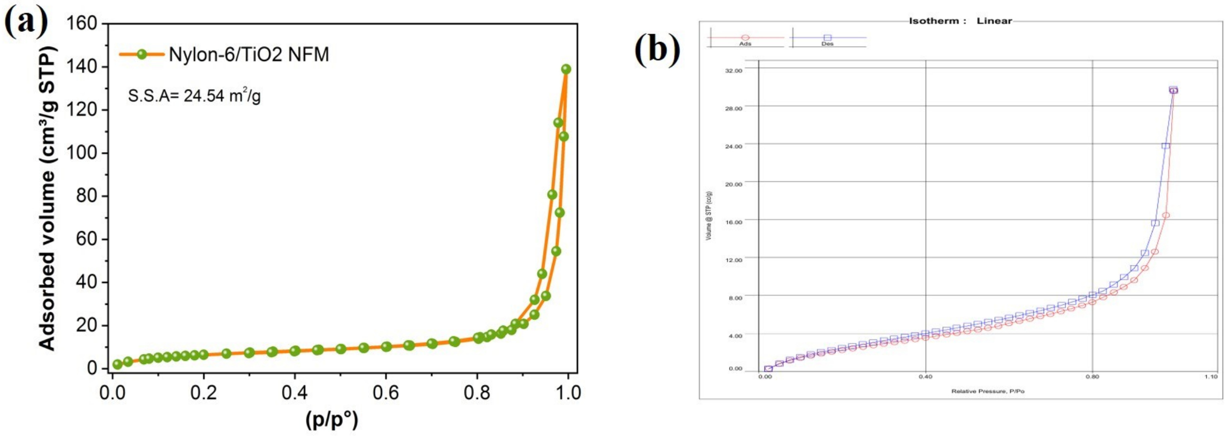

Fig. S3. BET surface area of (a) nylon-6/TiO_2 and (b) nylon-6 membranes, respectively.

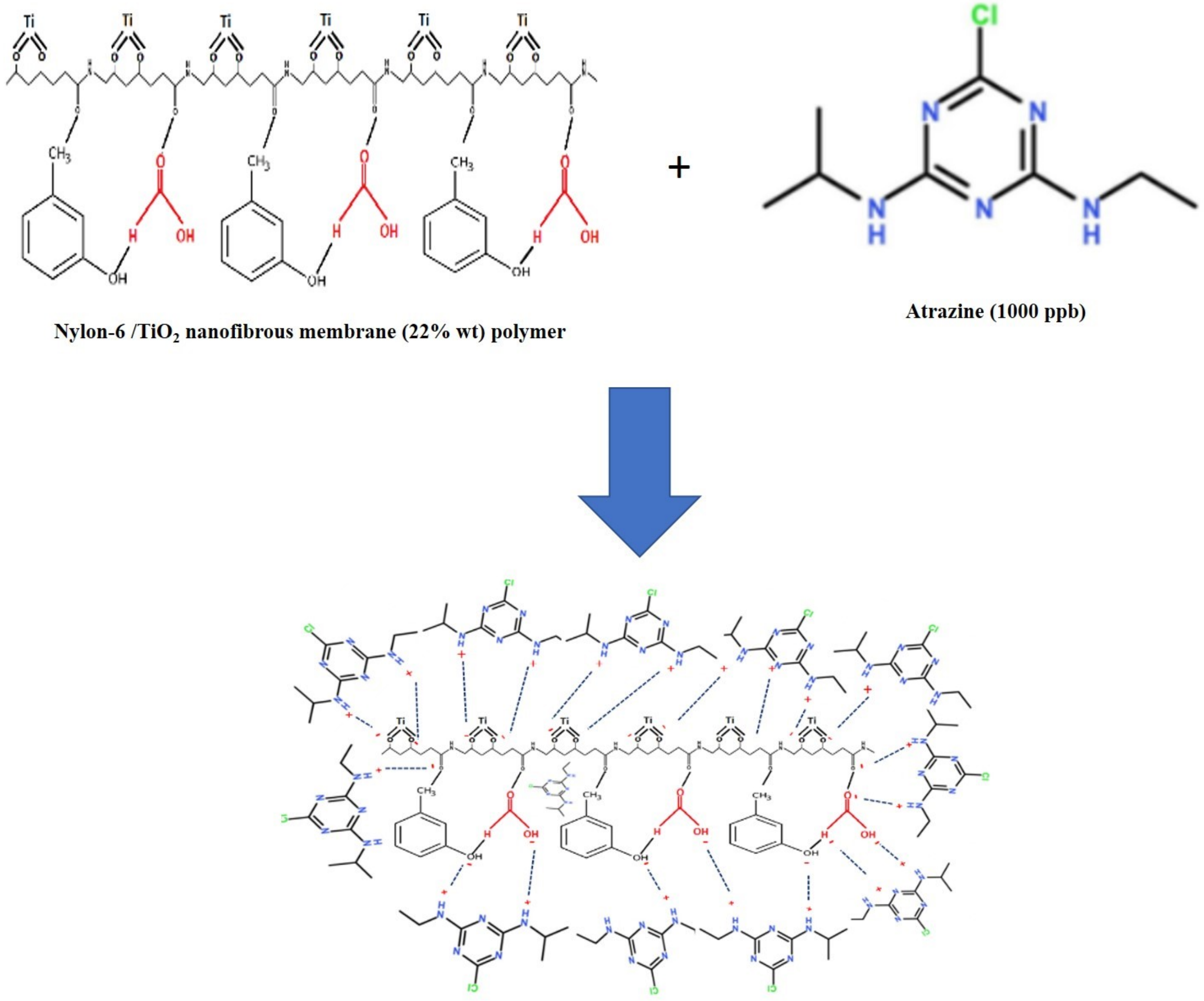

Fig. S4. Adsorption mechanism for the pollutant (atrazine) adsorption process by TiO_2-embedded nylon-6/TiO_2 nanofibrous membranes (22 wt%) polymer-derived nanofiber membrane.

Table S1. BET surface area, pore volume, and pore diameter of NFM

Sample	BET Surface Area (m^2/g)	Pore Volume (cm^2/g)	Pore Diameter (dv/d)
**Nylon-6**	10.124	0.045	5.042
**Nylon-6/TiO_2**	24.54	0.037	2.034
